# Teaching Youth & Families Self-Regulation Skills to Disrupt the Impact of Adverse Childhood Experiences: The THRIVE Study Protocol for a Randomized Controlled Trial

**DOI:** 10.21203/rs.3.rs-9046934/v1

**Published:** 2026-03-17

**Authors:** Brianna Johnston, Afsaneh Saghafi, Jayla Aldridge, Jenny Fotang, Mohammad Eghbal Heidari, Roxette Lopez, Aura Valencia, Sanghyuk Shin, Norweeta Milburn, Julian Thayer, Dawn Bounds

**Affiliations:** University of California, Irvine; University of California, Irvine; University of California, Irvine; University of California, Irvine; University of California, Irvine; University of California, Irvine; University of California, Irvine; University of California, Irvine; University of California, Irvine; University of California, Irvine; University of California, Irvine

**Keywords:** adverse childhood experiences, adolescents, self-regulation, substance use prevention, community health workers, biofeedback, family intervention, health inequities

## Abstract

**Background:**

Adverse Childhood experiences (ACEs) disproportionately affect minoritized and low-income youth. Those with four or more ACEs are particularly vulnerable to chronic, unpredictable stress, which shapes their stress perception and response. Higher exposure to ACEs is associated with an increased risk of self-dysregulation, early initiation of alcohol and substance use, and cardiometabolic issues, including disruptions in heart rate variability, sleep disturbances, weight dysregulation, and elevated blood pressure. While some evidence-based family interventions aimed at preventing traumatic stress exist, research explicitly focused on interventions using psychoeducation incorporating biofeedback remains limited. The purpose of this paper is to describe the study protocol for a randomized controlled trial with adversity-impacted adolescents ages 11 to 14 and their caregivers. The THRIVE Study examines the efficacy of the Garnering Resilience in Traumatized youth and families (GRIT) intervention, a psychoeducational coaching program focused on building buffering protective factors, compared to an attention control group receiving a grade-specific Digital Literacy Intervention in delaying the initiation of alcohol and cannabis use.

**Methods:**

Adolescent-caregiver dyads (n= 210) are recruited to participate in six 1-hour intervention sessions conducted over 8 weeks via Zoom, facilitated by an intervention-specific community health worker (CHW). In-person assessments are conducted at specific time points: pre-intervention, post-intervention, 6-month follow-up, and 12-month follow-up. During these assessments, heart rate variability using the emWave Pro Plus and survey data are collected using REDCap to evaluate emotional and cognitive function, and behavioral outcomes (e.g., sleep disturbances and substance use). Additionally, participants complete a booster session and online surveys at 6-months post intervention.

**Discussion:**

The THRIVE Study evaluates a psychoeducational health coaching intervention that incorporates biofeedback. The findings from this RCT have the potential to build buffering protective factors in adversity-impacted adolescents and their caregivers, reducing long-term health inequities among minoritized and underserved populations.

**Trial registration:**

trial registration name: ClinicalTrials.gov, trial registration number: NCT06821035, registration on date: July 18th, 2025

## Introduction

Adverse Childhood Experiences (ACEs), which include childhood maltreatment, family dysfunction (1–4), and other forms of trauma, constitute a serious public health issue in the United States, disproportionately affecting minority and impoverished populations (5–7). Approximately 60% of adults (8,9) and nearly half of adolescents in the U.S. report exposure to at least one ACE, and those exposed to four or more ACEs (heretofore referred to as adversity-impacted) face greater risks of emotional dysregulation and challenges in self-regulation (9–11). This heightened vulnerability often leads to maladaptive coping mechanisms, including the early initiation of alcohol and substance use (12).

The physiological consequences of ACEs are profound, as toxic stress can disrupt the body’s autonomic nervous system, leading to a chronic state of dysregulation (13,14). One way to measure and address this dysregulation is through vagally-mediated heart rate variability (HRV), which refers to the variation in time intervals between heartbeats and is widely accepted as a biomarker of autonomic function and self-regulation (15–17). HRV reflects the body’s ability to adapt to stress and manage emotional responses, making it a key indicator of how well individuals can regulate their physiological and emotional states (18). Lower HRV has been linked to increased stress, emotional dysregulation, and greater vulnerability to substance use (19–22). Given the malleability of HRV and its established association with stress, substance use, and mental health challenges, HRV biofeedback (HRV-BF) offers a promising approach to enhance self-regulation in adversity-impacted youth. HRV-BF, using tools like the Inner Balance^™^ Trainer, leverages techniques such as slow-paced breathing to strengthen vagal tone, reduce stress, and promote emotional resilience (15–17,23–25).

Building on these principles, the Garnering Resilience in Traumatized Youth and Families (GRIT) program was developed as a community health worker-led health coaching intervention to integrate HRV-BF with psychosocial support. The conceptual framework of GRIT is rooted in the Shift and Persist model, which posits that children who experience chronic, unpredictable stress develop altered perceptions of and responses to stress (26,27). However, specific strategies, referred to as Shift and Persist, can mitigate these effects. These strategies, facilitated through a community health worker (CHW) serving as a positive role model, teach adolescents to self-regulate their emotions through cognitive reappraisals (e.g., HearthMath techniques), build trust via co-regulated relationships, and adopt a future-oriented mindset by setting goals, building healthy habits, and clarifying values.

The GRIT program also aligns with the California Surgeon General’s guidelines for mitigating toxic stress by promoting key factors, including nurturing relationships, mindfulness, and healthy habits (28). These theoretical foundations shape GRIT’s comprehensive approach to facilitating self-regulation in adolescent-caregiver dyads, mitigating the effects of toxic stress by building buffering protective factors, and preventing the early initiation of substance use in adversity-impacted youth.

The purpose of this paper is to describe the protocol of the Teaching Healthy Regulation in Individuals and Vulnerable Environments (THRIVE) study, which evaluates the efficacy of the GRIT program in a randomized controlled trial (RCT). The active control condition was designed to match the intervention in duration and facilitator contact while avoiding components targeting self-regulation or substance use prevention. This secondary prevention intervention integrates HRV-BF technology within CHW-facilitated health coaching to enhance self-regulation in adversity-impacted adolescent-caregiver dyads. By collecting objective data on biopsychobehavioral indicators of self-regulation and toxic stress, the THRIVE study aims to further our understanding of how interventions like GRIT can mitigate the effects of ACEs and prevent the early initiation of substance use.

## Methods

### Study Design and Study Aims

The THRIVE Study is a two-armed RCT designed to evaluate the effects of GRIT, a psychoeducational health coaching intervention aimed at preventing the early initiation of regular alcohol and cannabis use in adversity-impacted adolescents ages 11 to 14 who are at high risk for toxic stress. This study will include two groups: an intervention group receiving the GRIT intervention and an active control group receiving a digital literacy curriculum. The study will assess the impact of the GRIT intervention on self-regulation and substance use outcomes through repeated measures at pre-intervention, post-intervention (2 months post-baseline), 6-month follow-up (8 months post-baseline), and 12-month follow-up (14 months post-baseline).

There are two specific aims for this study: (1) test the efficacy of GRIT on preventing the early initiation of regular alcohol and cannabis use among adversity-impacted adolescents ages 11–14 years who do not regularly use these substances at baseline; and (2) test the mediating effects of youth and caregiver self-regulation on the relationship between the GRIT program and adolescent substance use outcomes. A digital literacy active control group will allow for comparison in youth and caregiver outcomes. We hypothesize that adolescents who receive GRIT will have lower rates of regular alcohol and cannabis use compared to those in the active control group post-intervention, and that youth and caregiver self-regulation will mediate the relationship between the GRIT intervention and youth outcomes.

### Ethics Approval

Ethics approval was obtained from the University of California, Irvine Institutional Review Board (IRB; #4821). Caregivers provide written informed consent, and adolescents provide assent before participation. Consent and assent are obtained by trained research staff in accordance with IRB approval. Important protocol changes will be submitted to the IRB for approval prior to implementation. Approved changes will be updated to the trial registry. The study poses minimal risk to participants. We do not expect to see any detrimental risks or discomforts arising as a result of this health coaching intervention. Nevertheless, participants may experience emotional or psychosomatic reactions from sensitive information discussed, reported, or described. To ameliorate this, we provide a mental health resource guide at the first in-person assessment. Adolescents may feel a sense of embarrassment about seeking help, and caregivers may feel embarrassment about possibly being perceived as a “bad caregiver” or may feel a sense of stigma regarding the benefits of mental health. To address this, we will instruct the community health workers (CHWs) to validate and normalize healthy help-seeking behaviors, as well as unpack any stigmatizing feelings toward the intervention itself. No financial compensation for trial-related harm or additional post-trial care is anticipated.

### Patient and Public Involvement

The GRIT intervention was developed based on the literature on evidence-based strategies, previous family-based interventions (e.g., STRIVE), and the California Surgeon General’s recommendations. Patients were not involved in the design or conduct of the study, however health sciences students who served as research assistants and health coaches in the pilot study and community health workers assisted with study design and implementation.

### Power and Sample Size

We used the General Linear Mixed Model Power and Sample Size (GLIMMPSE) application to estimate the sample size needed for this study using the approach for longitudinal data (29). We calculated the sample size needed to detect a 40% lower change in use across time among the GRIT versus active control arm for both outcomes, as the Strengthening Families Program cited 50% reductions (30). In addition, we judged that a 40% reduction in initiation rate would be needed for a clinically meaningful effect. Given that exposure to four or more ACEs is associated with a two-to three-fold increase in early initiation (i.e., before age 14) of alcohol and cannabis use and national high school samples report initiation of alcohol before 13 to be 15% and cannabis to 5%, we conservatively assumed a 20% initiation for alcohol and 8% for cannabis for our high-risk study population (31–34). We also accounted for 20% attrition during the 14-month follow-up period and a variance inflation factor of 1.4 to accommodate the mediation analysis. Under these assumptions, a sample size of 105 dyads per arm (total N = 210 dyads) would be needed to provide 80% power at a nominal alpha of 0.025 for each outcome (adjusted for multiplicity to achieve an overall alpha of 0.05 for both outcomes). Previous research, such as the Support to Reunite, Involve, and Value Each Other (STRIVE) study and its adaptation, STRIVE +, has informed the sample size for this study. STRIVE evaluated a 5-session dyadic psychoeducational intervention with youth experiencing homelessness, enrolling 151 youth with a retention rate of 77% at a follow-up (3, 6, and 12 months) (35). STRIVE demonstrated efficacy in significantly reducing substance use, sexual risk, alcohol use, and illicit drug use over 12 months compared to the control condition. Given these findings, family based-interventions like GRIT may similarly reduce substance use and improve overall health and well-being. The findings from STRIVE+ further informed the design of our study, particularly highlighting the need for additional sessions and booster sessions. In STRIVE+, parents requested more sessions to practice newly acquired skills, as they experienced difficulties with familial connection. Based on STRIVE’s successful retention and outcomes, we believe that a sample size of 210 dyads (N=420) for the GRIT study is reasonable, as it allows us to detect meaningful differences in substance use outcomes between the GRIT intervention and the control group.

### Sampling

Two hundred and ten adolescent-caregiver dyads (N=420), with adversity-impacted youth aged 11–14, will be recruited. Adolescents are considered adversity-impacted if they score in the high-risk category (4 or more ACEs or any number of ACEs and an ACE-related condition [e.g., school difficulties, symptoms of anxiety and/or depression]). Youth will be recruited from schools, health clinics, and youth-serving community organizations in Southern California. Eligible participants will be randomly assigned to either the GRIT intervention or the active control intervention (i.e., Digital Literacy).

Inclusion criteria for youth include: 1) being aged 11–14 years and scoring in the high-risk category on the ACEs and Toxic Stress Risk Assessment Algorithm; 2) having access to a smartphone and willingness to download an app if randomized to the GRIT intervention; and 3) have a positive, supportive adult such as a parent/guardian/primary caregiver aged 18 years or older who is English or Spanish-speaking and willing to participate in the intervention. The inclusion criteria for caregivers require them to be willing to engage in the intervention alongside the youth participant.

Exclusion criteria for youth participants include: 1) reporting alcohol or cannabis use within the last 14 days; 2) currently being enrolled in another family-based intervention (e.g., family therapy); 3) being in acute distress and requiring immediate care (e.g., imminent risk of harm to self or others, active psychosis); 4) requiring supervised visits by the Department of Children and Family Services (DCFS) with the identified caregiver; and 5) cases where the parent/guardian/primary caregiver declines participation, in which case the family will referred to other community-based programs.

### Recruitment Strategy

Black and Hispanic/ Latinx populations in California are more likely to have experienced 4 or more ACEs (32). As such, we will ensure that youth who self-identify as Black or Hispanic/ Latinx are well-represented in our sample by recruiting from diverse settings across Southern California. Recruitment for this study will leverage established relationships with schools, health care providers screening for Adverse Childhood Experiences (ACEs), especially in federally qualified health and school-based health centers, and community organizations serving adversity-impacted youth. While participant enrollment will come from these strong partnerships, the entire research team will also continue to foster relationships with additional schools and youth-serving organizations through community service (e.g., volunteering, board service), educational workshops, and tabling at community events.

### Recruitment Procedures

The research team will enroll each dyad in a staggered and overlapping manner. This strategy ensures that some participants will be in the consent and data collection process while others progress through various stages of the intervention. Enrolling at a rate of 4–8 dyads/month will allow us to reach our recruitment goal of 210 dyads. To inform potential participants, an IRB-approved flyer describing the study will be distributed to both the youth and their caregivers by our community partners. A scannable QR code linking to an online interest and eligibility form on REDCap will be provided for dyads to express their interest in the program. This form will collect contact and eligibility information to allow the research team to follow-up, share more information about the study, and confirm eligibility. The research team will use the Pediatric ACEs and Life Event Screener (PEARLS) to assess if the adolescent qualifies for the high-risk category based on their ACE score. PEARLS collects data on the number of ACEs the adolescent has experienced and identifies any recent ACE-associated conditions. Following eligibility screening, informed assent will be obtained from the youth and informed consent from the youth’s legal guardian (for youth participation) and the caregiver before enrollment. Participants will be given up to 72 hours to ask questions and decide about their participation. Once eligibility is confirmed, the baseline assessment will be scheduled, where the informed consent process will be finalized.

### Randomization

Eligible participants will first be stratified by ethnicity and ACEs scores, and then randomly assigned to one of the two study groups (GRIT Intervention or Digital Literacy Active Control) using a 1:1 ratio. Randomization will be performed using the REDCap randomization module, which will facilitate the computerized random allocation of dyads. Prior to study initiation, random allocation tables will be generated based on ethnicity and ACEs score (1–3 vs. 4 or more) using a custom R script. All research staff will remain blind to the randomization tables, which will be uploaded into REDCap for automatic allocation of dyads to study arms. After the baseline assessment, the research team will notify the project coordinator, who will verify the random assignment in REDCap.

### Enrollment Procedures

Once participants, both youth and caregiver, are jointly identified as eligible by the initial screener form, both caregiver and youth are automatically emailed a response through the REDCap system. This email contains instructions about the participant’s next steps for enrollment, as well as a link to a Bookings page where they may select a date for their initial baseline assessment. Within 48 hours of a confirmed eligible screening, the assessment team will use a phone/email script to either confirm a date, if provided in the screener, or set a date for the initial baseline assessment. Once both date and time are determined, the assessment team will help the interested dyad identify a quiet and accessible location (e.g., university space, home, library, local center) at which the assessment will be held. The assessment team then sends out confirmation for the baseline assessment via email and text message. This confirmation contains the location, date, time, and essential reminders for them not to eat a heavy meal, exercise, or drink caffeine or other stimulants 60–90 minutes before the assessment in preparation for the heart rate variability (HRV) assessment. This email will also contain two versions of the consent form, one in English and one in Spanish, for review at least three days prior to the baseline assessment. Two more reminder emails will be sent the day prior to the assessment and the morning of the baseline assessment with the same content as the initial confirmation email, confirming the appointment. Official enrollment begins at the baseline assessment. Once the dyad arrives at the designated location for their appointment, the enrollment team guides the dyad through the informed consent form and verbally quizzes both participants on sections detailing timeline, risk, and data privacy to ensure a thorough understanding of what their assent/consent and participation in the study entail. Once participants express a thorough understanding of the study and electronically sign the consent forms, the assessment procedures, including an HRV assessment, biometric measurements, and various self-report questionnaires, begin. In order to randomize the dyad into one of the two arms of the study, data from the youth’s initial screener and demographics survey will be extracted for randomization. When randomization occurs, dyads are officially enrolled into either the Digital Literacy or the GRIT arm of the study and given the corresponding materials for that program. Dyads are not blinded from their assignment into one of the two interventions; their assigned health coaches, however, are blinded to all collected data during the dyad’s assessments.

### Retention Strategies

#### Incentives.

Dyads receive a monetary incentive for completing their data collection measures. Both the youth and caregiver are compensated with a $50 Amazon e-gift card for completing each assessment at baseline, post-intervention, 6-month post-intervention, and 12-month post-intervention. If dyads are unable to complete an in-person data collection where we conduct the HRV assessment and collect anthropometrics, but at least complete the online surveys, they receive a $25 Amazon e-gift card. Additionally, a monthly competition is held for dyads randomized to the GRIT arm, in which individuals and dyads with the highest average of minutes practiced using their Inner Balance trainer receive a bonus $10 Amazon e-gift card. This bonus reward is to encourage engagement in the self-regulation techniques using biofeedback outside of the intervention session time.

#### Reminders.

A variety of contact information is obtained from the youth and caregiver, to ensure that contact is maintained throughout the 14-month study period. Phone number, e-mail, mailing address, social media handles, and emergency contact information are collected in addition to preferred methods of contact. Dyads receive both emails and text message reminders the day before and day of data collection assessments and intervention sessions.

#### Mobile Assessments.

Dyads are given the opportunity to choose the time and location for their data collection visit, such as their homes and local public spaces. To be considerate of work and school schedules, evenings and weekends are included as possible appointment options for assessments to occur. With the exception of the baseline assessments, dyads are also able to access and complete the online surveys ahead of their in-person assessment for the duration of the in-person visit. There is no in-person visit for the 6-month post-intervention assessment.

### Format and Session Delivery

Sessions are approximately 60 minutes in length and delivered weekly over an eight-week period. Over the course of 8 weeks, families participate in six core sessions once a week, followed by a booster session approximately six months later. Sessions are designed to be delivered online in a three-person interactive model involving a youth-caregiver dyad and a trained Community Health Worker (CHW). A bilingual CHW is available to conduct each session in English and/or Spanish. Each session is conducted live via a secure video conferencing platform and lasts approximately 60 minutes. Sessions include interactive lesson slides, real-world scenarios, and worksheets that are completed in real time during the session. These in-session activities are designed to reinforce core concepts and support the development of healthy habits. Each session concludes with a lesson review. The lesson review is led by the CHW, assessing if the dyad understood the content and providing space to clarify any questions on the concept.

Each session is facilitated by a Community Health Worker (CHW) trained in adolescent engagement and the delivery of structured, skills-based interventions. Using a standardized lesson plan guide and slides. The CHW guides the dyad through each lesson, encouraging real-time participation using verbal responses and worksheet activities. The materials are written at a middle school reading level and available in both English and Spanish. CHWs foster a safe and supportive environment that encourages honest discussion, while avoiding being clear about their role as a coach and not a therapist.

### Adherence and Fidelity Procedure

Adherence and fidelity are monitored through multiple methods to maintain consistent delivery and meaningful engagement in each session. CHWs complete a structured adherence checklist after every session, documenting session completion, participant attendance, in-session worksheet, and lesson review completion. At the conclusion of each session, the youth-caregiver dyad is asked to complete a post-session survey designed to assess session quality, coaching effectiveness, and perceived impact. This survey includes Likert-scale items that explore how helpful the session was and how well the CHW facilitated discussion. These responses help monitor the fidelity of the coaching relationship and the content delivery.

Periodic observations by the project coordinator further support quality assurance, allowing for feedback for CHWs. A minimum of 10 percent of CHW’s recorded coaching sessions are watched and reviewed by the program coordinator to ensure fidelity. The coordinator meets regularly with CHWs to discuss progress with dyads, room for improvement, and ways to resolve any arising conflict between youth and caregiver. The project coordinator and CHWs also collaborate to ensure dyad retention within the program if notable barriers to attending sessions are observed. In addition, trial oversight is maintained by the data management team that includes the principal investigator, project coordinator, and co-investigator (i.e., statistician) through weekly review of enrollment progress and protocol adherence in accordance with IRB requirements.

### Intervention Group: Garnering Resilience in Traumatized Youth and Families

Participants randomized to the intervention group will receive GRIT, a six-session psychoeducational health coaching intervention designed to promote self-regulation and healthy habit development. GRIT is delivered to adolescent–caregiver dyads using a trauma-informed, coaching-based approach that emphasizes responsive communication and skill practice. Adolescents and caregivers may attend sessions jointly or individually, as needed.

To support skill acquisition and ongoing practice, each dyad is provided with an Inner Balance Trainer made by HeartMath^®^ and instructed in heart rate variability biofeedback (HRV-BF) practice. CHWs introduce and reinforce HRV-BF throughout the intervention, and regular practice using HRV-BF is incentivized through monthly competitions based on the total number of minutes practiced. Participants retain the device following the study’s completion to support continued use.

#### Content.

GRIT integrates components from Trauma-Focused Cognitive Behavioral Therapy (TF-CBT), Acceptance and Commitment Therapy (ACT), and the Shift and Persist framework. The curriculum emphasizes psychoeducation and skills to enhance self- and co-regulation, stress management, communication, and resilience. Core content includes education on toxic stress and Adverse Childhood Experiences (ACEs), emotion identification, diaphragmatic breathing, heart rate variability biofeedback (HRV-BF), cognitive reframing, values clarification, and goal setting. Later sessions focus on the California Surgeon General’s recommendations for preventing toxic stress through healthy habit formation (e.g., nutrition, sleep, and positive relationships) and communication strategies that support responsive caregiving and family functioning.

This intervention is delivered by community health workers (CHWs) who completed a trauma-sensitive HeartMath^®^ certification course called Resilient Heart^™^ and training on the GRIT intervention protocol prior to participant enrollment. The GRIT training includes a minimum of 10 weeks of training on foundational GRIT concepts, including 10 self-study modules delivered as an online course. Each CHW is also required to develop their own self-care practice, which includes applying concepts of Cognitive Behavioral Therapy (CBT) and Acceptance and Commitment Therapy (ACT) to their own lives as well as practicing self-regulation techniques using HeartMath’s Inner Balance Trainer. Each CHW completes practice sessions to demonstrate their coaching skills and receive structured feedback. The CHW delivering the digital literacy control receives separate training and attends separate supervision meetings to prevent contamination.

CHWs then support adolescents and caregivers integrating these skills into daily routines, with an emphasis on modeling and reinforcing co-regulation strategies within the home. The intervention concludes with individualized goal setting and maintenance planning to promote sustained resilience. Approximately six months after completion of the core intervention, families are invited to participate in a booster session to reinforce key concepts, re-engage in HRV-BF practice, and reflect on progress. The booster includes a brief review of emotion regulation skills, breathing and HRV-BF techniques, and a discussion of how GRIT strategies have been applied in daily life to support continued practice and maintenance of self- and co-regulation gains.

### Comparison Group: Digital Literacy

The Digital Literacy Program (DLP) serves as an active comparison group in this study. The program is based on lesson content sourced from Common Sense Media’s Digital Citizenship curriculum (Common Sense Education, n.d.). The curriculum offers a structured, interactive, grade-specific curriculum guide to help youth ages 11 to 14, and their caregivers build essential skills for navigating today’s digital world. The program focuses on topics such as media balance, media literacy, online safety, and responsible technology use. The DLP matches the intervention group in duration, CHW facilitation, and level of participant interaction. Providing an active control condition centered on digital behaviors and family engagement.

#### Content.

The DLP curriculum covers six key digital literacy topics, including digital habits, online privacy, digital footprint, digital communication, misinformation, media literacy, and safety. Each session includes a behavior change activity, such as screen time reflection and media balance commitments. These activities are included directly in the lesson and completed during the session. The worksheets guide families through discussions, goal-setting exercises, and practical applications of the core lesson.

### Discontinuation Criteria

Study participants may discontinue participation at any point during the study. The intervention may be discontinued for a participant if safety concerns arise that limits the participant’s ability to continue participation such as psychological distress (e.g. imminent risk of harming self or others), or withdrawal of consent or assent. Unless participants request complete withdrawal, data collected prior to discontinuation will be used for data analysis in accordance with IRB policy.

### Outcomes

The primary outcome is adolescent initiation of regular alcohol and cannabis use. Alcohol and cannabis use will be measured using items from the CDC Youth Risk Behavior Survey (YRBS) and the frequency of use will be assessed using the Timeline Followback (TLFB) method. Regular alcohol or cannabis use is defined as at least weekly use. Secondary outcomes include anthropometrics, heart rate variability, emotional regulation, psychological health, health behaviors, and family functioning. Outcomes will be measured at baseline, post-intervention (2 months), 6 months post-intervention, and 12 months post-intervention.

### Measures

Over the duration of the study, participating youth and caregivers are invited to complete data collection assessments at four timepoints: pre-intervention (baseline), post-intervention (2 months post-baseline), 6-months post-intervention (8 months post-baseline) & 12-month post-intervention (14 months post baseline). Assessments consist of a set of self-report questionnaires and objective measures of heart rate variability (HRV). All questionnaires are completed online through REDCap, a secure database platform. Themes covered in questionnaires include youth and caregivers’ individual substance use and health behaviors, family functioning, and psychological and emotional health. All self-report measures have demonstrated good reliability, including internal consistency and moderate to good test-retest reliability, in previous research with adolescents and adults (36–44). HRV measures are collected directly from the youth and caregiver by the assessment team at three time points: pre-intervention, post-intervention, and 12-month post-intervention.

### Anthropometrics

Anthropometric measures collected from youth and caregiver include height, weight, waist circumference, pulse, and blood pressure.

### Heart Rate Variability (HRV)

HRV is an index of objective physiological measures of stress (18). It is collected using a heart rhythm monitoring system called emWave Pro Plus. The system was developed by the HeartMath Institute, a leader in developing HRV biofeedback technology (45). The software collects real-time HRV data from a non-invasive earlobe clip that is sensitive to heart rate and allows data input to be tracked on a computer. The device is used to capture participants’ HRV in 3 different states: resting (3 minutes), stress prep (3 minutes), and deep breathing (1minute). Changes within an individual’s HRV over time are examined, with higher HRVs reflecting an individual’s ability to quickly return to homeostasis and a more regulated nervous system (46)

### Substance Use

Alcohol, cannabis, and other substances’ use are collected from youth using select multiple-choice items from the CDC Youth Risk Behavior Survey (YBRS) questionnaire that ask about substance use history, frequency, and age of onset. Positive reporting of alcohol or cannabis use by youth prompts a Timeline Followback Method Assessment (TLFB) in which youth are asked to recall and report the estimated amount of consumption across the last 30 days from the day of assessment (47,48). Caregiver’s alcohol and cannabis use is measured using the Alcohol Use Disorders Identification Test (AUDIT-C) and Cannabis Use Disorder Identification Test (CUDIT-R), which assess frequency and amount of substance consumption in the past year (36,40).

### Emotional Regulation

Difficulties in Emotional Regulation- Short Form (DERS-SF) is used to collect data on emotional regulation abilities for both youth and caregivers. The DERS-SF is an 18-item self-report, an abbreviated version of the original 36-item questionnaire (49). Emotional regulation is measured on 6 subscales: strategies, non-acceptance, impulse, goals, awareness, and clarity. Total scores range from 18 to 90, with higher total scores on the DERS-SF indicating greater difficulties in emotion regulation.

### Psychological Health

Psychological flexibility was measured for youth participants using the Acceptance and Fusion Questionnaire (AFQ_Y8)(39). The Acceptance and Action Questionnaire (AAQ-II) was used to measure psychological flexibility in adults. Higher total scores on the AFQ_Y8 and AAQ-II are indicative of lower psychological flexibility.

We measured levels of perceived stress for youth and caregivers using the Perceived Stress Scale (PSS-10). The 10-item questionnaire evaluates stress according to two subscales: perceived helplessness and lack of self-efficacy. A higher overall score is indicative of higher levels of perceived stress.

### Health Behaviors

Nutrition and physical activity amongst youth participants were collected using select multiple-choice items from the CDC Youth Risk Behavior Survey (YBRS) questionnaire. Items asked about the frequency of engagement in physical activity and nutrition, such as the inclusion of food items in the diet (i.e., fruit, green salad, and other vegetables). The Pittsburgh Sleep Quality Index (PSQI) was used to collect data on youth and caregiver sleep behaviors. The PSQI is a 19-item self-report questionnaire that assesses seven component scores: subjective sleep quality, sleep latency, sleep duration, habitual sleep efficiency, sleep disturbances, use of sleep medication, and daytime dysfunction (50). PSQI global scores range from 0 to 21, with higher scores than 5 indicating sleep disturbance.

### Family Functioning

The Family Assessment Device was used to examine family functioning reported by youth and caregivers. The 60-item self-report questionnaire collects data on families’ ability to problem solve, communicate, have distinguished roles, control behavior, and affective response and involvement. Higher overall scores indicate worse levels of family function (42)

### Data Management

A data safety monitoring board will be appointed for this study by month 4. Our board will be composed of five members: three external members with experience working with the population and two senior investigators from our institution who conduct behavioral research with similar populations. They will meet a minimum of two times per year and maintain and approve minutes of meeting. The NIH PO will be kept informed regarding changes to the membership of the DSM board. Data will be reviewed as every 30 participants enters the study. We will conduct interim analysis of safety endpoints to identify possible changes in protocol needed to improve safety. Given the nature of this intervention, traditional medical and clinical trial stoppage rules are not appropriate. Therefore, the DSMB and the PI will make determinations as to whether the trial should be halted in the unlikely event of serious adverse events.

All study data are managed using the secure, HIPAA-compliant Research Electronic Data Capture (REDCap) system hosted by the University of California, Irvine (51,52,52). Screening, randomization, intervention monitoring, and follow-up assessments are maintained in REDCap projects to streamline workflow and maintain participant confidentiality. Access to the project is role-restricted, and all user activity is automatically audited. Youth and caregivers complete their questionnaires independently through individualized REDCap survey links, and branching logic ensures they only view items relevant to their age and role. HRV recordings and anthropometric data are collected by trained assessment staff and entered directly into standardized electronic case report forms with embedded range checks, logic constraints, and mandatory fields to minimize entry errors.

Data quality is maintained through multiple layers of verification. Weekly automated reports flag missing values, inconsistent responses, or out-of-range entries, which are subsequently reviewed and resolved by the Data Manager through a structured query process. In addition, semi-annual data audits verify approximately ten percent of all records against source documents, including fidelity logs, HRV raw files, and attendance records, to ensure accuracy and completeness. All participant data are stored under a unique study identification number. All data will be retained for at least seven years after study completion in accordance with institutional and federal requirements. Only de-identified, analysis-ready datasets will be exported for statistical analyses.

### Data Analysis Plan

Baseline characteristics will be summarized using descriptive statistics to assess group balance. Primary analyses will follow the intention-to-treat principle, with all randomized participants included. Changes in adolescent alcohol and cannabis use will be compared between study arms using generalized linear mixed models (GLMMs) to account for within-subject correlation over time. Alcohol and cannabis use will be dichotomized as no or occasional use versus regular use (weekly or more) to evaluate the intervention’s effect on preventing initiation of regular substance use. Statistical significance will be assessed using two-sided tests with an alpha level of 0.05.

Multilevel mediation analyses will be conducted to examine mechanisms underlying intervention effects, with youth and caregiver emotional regulation specified as primary mediators. Additional exploratory mediators will include heart rate variability, psychological and cognitive flexibility, youth health behaviors (HRV biofeedback practice, sleep, nutrition, physical activity), and environmental factors (parental monitoring, family functioning, and caregiver substance use). Indirect and direct effects will be estimated using residual-based bootstrap methods to generate 95% confidence intervals. Sex differences will be evaluated in secondary analyses by including a sex-by-intervention interaction term.

Our primary analysis will be performed using mixed effects analysis, which provides unbiased estimates when missing data is missing at random (MAR). We will also consider multiple imputations to handle missingness using the Rubin method implemented in R package mice (53,54).

### Dissemination Plan

Findings from this trial will be disseminated through publications in interdisciplinary journals and presentations at professional national or international conferences. Trial findings will be reported on ClinicalTrials.gov in accordance with reporting standards. Upon completion of the study, aggregate findings will be shared with stakeholder workshops or community engagement centers. All the final peer-reviewed journal manuscripts that arise from this proposal will be submitted to Pubmed Central in accordance with NIH Public access policy.

## Discussion

Adverse Childhood Experiences disproportionately affect minoritized and low-income youth, with those experiencing four or more ACEs at substantially elevated risk for emotional dysregulation and early substance use initiation (2,3). Early substance use before age 14 is particularly concerning, as it increases the likelihood of developing substance use disorders sixfold (55). Despite the well-documented consequences of ACEs, research on interventions integrating psychoeducation with biofeedback technology for prevention of substance use in adversity-impacted youth remains limited (56,57). The THRIVE study addresses this critical gap by evaluating a novel intervention that combines heart rate variability biofeedback (HRV-BF) with family-based psychoeducation delivered by community health workers.

### Rationale for Key Design Decisions

Heart rate variability, the variation in time intervals between heartbeats, serves as a biomarker of autonomic nervous system function and self-regulation capacity (59). Toxic stress from ACEs disrupts autonomic regulation, leading to chronic sympathetic activation, reduced parasympathetic activity, and decreased HRV(60). This physiological dysregulation impairs emotional and behavioral self-regulation, increasing vulnerability to substance use. Given HRV’s malleability, HRV-BF training using the Inner Balance^™^ Trainer offers a promising intervention approach. By employing paced breathing techniques with real-time visual feedback, HRV-BF strengthens vagal tone and promotes physiological resilience (15–17).

The GRIT intervention integrates this biofeedback technology within a comprehensive psychoeducational framework grounded in the Shift and Persist model (26), incorporating elements from Trauma-Focused Cognitive Behavioral Therapy and Acceptance and Commitment Therapy (61,62). This combination addresses both biological (autonomic dysregulation) and psychological (emotion regulation, coping skills) pathways through which ACEs contribute to substance use risk. The dyadic approach, simultaneously targeting adolescent and caregiver self-regulation, promotes co-regulation within the family system and may interrupt intergenerational transmission of trauma responses (63–65), a strategy supported by evidence demonstrating enhanced efficacy of parent-involved interventions.

The community health worker delivery model enhances cultural concordance, accessibility, and potential scalability in underserved communities compared to clinician-delivered interventions (66). Task-shifting to CHWs addresses workforce shortages and institutional barriers, including clinicians’ high workload and limited time during regular visits (67). The active control condition (Digital Literacy Program) matches the intervention in duration, CHW facilitation, and participant interaction while controlling for attention effects and providing meaningful benefits to all participants (68,69).

### Methodological Strengths

The study protocol has several notable strengths. The rigorous randomized controlled trial design with stratified randomization ensures group balance and allows strong conclusions about intervention efficacy. The multi-method assessment approach combines objective physiological measures (HRV via emWave Pro Plus, anthropometrics) with validated self-report questionnaires across multiple domains, providing a comprehensive evaluation of intervention effects (14,15,68). The longitudinal design with 14-month follow-up allows assessment of sustained intervention effects beyond the immediate post-treatment period, addressing the challenge of behavioral relapse often observed in short-term interventions (70). Multiple retention strategies, including flexible scheduling, virtual delivery via Zoom to reduce transportation barriers, incentives ($400 per dyad), and booster sessions, are implemented to minimize attrition in this vulnerable population (71,72).

### Practical Challenges and Limitations

Several operational challenges are anticipated across recruitment, retention, intervention delivery, and data collection. Recruitment of adversity-impacted families who may distrust research requires strong community partnerships with trusted organizations, CHW involvement from the recruitment phase, and clear communication of potential benefits (73,74). Reaching minoritized populations disproportionately affected by ACEs is addressed through targeted recruitment from diverse settings across Southern California, bilingual materials and CHWs, and cultural tailoring of recruitment approaches and intervention delivery. The requirement for dyad participation may exclude some adversity-impacted youth lacking available caregivers, though flexible scheduling (evenings/weekends), virtual delivery options to reduce transportation barriers, and clear value propositions for both adolescents and caregivers aim to maximize participation.

High attrition in adversity-impacted populations is mitigated through multiple strategies: strong CHW-dyad relationship building, incentive structure ($400 per dyad total), multiple contact methods with proactive outreach, and flexible assessment locations including homes, community spaces, and university settings. Maintaining engagement over the 14-month study period is supported by regular contact between formal assessments, a booster session at 6 months post-intervention, remote survey completion options to reduce in-person burden, and monthly Inner Balance practice competitions for the GRIT group participants.

Intervention of fidelity across multiple CHWs is ensured through standardized training protocols, detailed intervention manuals and session guides, and ongoing supervision. Adherence and fidelity monitoring includes audio/video recording of all sessions, structured CHW-completed adherence checklists, post-session participant surveys assessing quality and CHW effectiveness, periodic observations by program leads, and regular fidelity review meetings (75,76).

Data collection presents specific challenges. Collecting sensitive data on substance use and trauma history is managed through confidential independent surveys where youth and caregiver responses remain private from one another, REDCap’s secure platform with role-restricted access, and trauma-informed assessment approaches. Objective HRV measurement in community settings requires standardized protocols to minimize measurement error, pre-assessment instructions (avoiding heavy meals, caffeine, and exercise), quiet controlled environments, and trained assessment staff with quality control procedures. Self-report bias in substance use assessment, a concern given the stigma surrounding adolescent substance use (77), is mitigated through validated measures and emphasis on confidentiality to encourage honest reporting.

Safety monitoring procedures include regular check-ins during intervention sessions, assessment of youth emotional distress and mental health at each timepoint, and clear protocols for responding to disclosures of abuse, suicidality, or acute distress. Established referral pathways include resource guides with mental health services provided at the baseline assessment, emergency referral protocols, and documentation of all safety concerns and actions taken.

Study limitations include the geographic focus on Southern California, which may limit generalizability to other regions. English/Spanish-only materials may exclude other linguistic groups. Technology requirements (smartphone access, digital literacy) may exclude some families despite the provision of Inner Balance devices and technical support. The 12-month follow-up period, while longer than many prevention trials, may not capture longer-term substance use trajectories, though it balances feasibility with assessment of sustained effects. The requirement for caregiver participation excludes youth without available caregivers, potentially missing some of the highest-risk adolescents.

### Significance, Impact, and Conclusion

The THRIVE study protocol presents a rigorous evaluation of an innovative intervention addressing the critical public health challenge of ACEs and early substance use initiation in vulnerable youth populations. This study represents the first intervention to rigorously evaluate HRV biofeedback integration within family-based psychoeducation for adversity-impacted youth, targeting both biological (autonomic regulation) and psychological (emotion regulation, coping skills) mechanisms underlying ACEs-related health risks.

If efficacious, GRIT offers a scalable, community-deliverable intervention addressing a critical gap in prevention services for populations bearing disproportionate adversity burden (3,56,66,67). The CHW-delivery model provides a sustainable and cost-effective approach readily integrated into existing community-based programs, schools, and healthcare settings (78), with potential for broader dissemination beyond specialized clinical environments. The intervention’s focus on preventing substance use initiation before age 14 has the potential to reduce long-term health inequities and associated healthcare costs (35,56). By targeting both biological (autonomic regulation via HRV-BF) and psychological (emotion regulation, coping skills) mechanisms, GRIT may offer advantages over traditional talk-therapy approaches alone.

The dyadic, family-centered approach addresses not only individual youth outcomes but also caregiver self-regulation and family functioning, potentially interrupting intergenerational cycles of adversity. Findings will advance understanding of mechanisms through which biologically-informed interventions prevent substance use in high-risk populations and inform refinement of future prevention efforts. If demonstrated effectively, the intervention may represent a replicable model suitable for implementation across diverse settings to reduce poor health outcomes in vulnerable populations.

## Supplementary Material

This is a list of supplementary files associated with this preprint. Click to download.

• Spiritchecklist.dotx

## Data Availability

Data will be shared using the National Addiction & HIV Data Archive Program (NAHDAP) repository within the Inter-university Consortium for Political and Social Research (ICPSR) platform.
